# Fabrication of Soft Tissue Scaffold-Mimicked Microelectrode Arrays Using Enzyme-Mediated Transfer Printing

**DOI:** 10.3390/mi12091057

**Published:** 2021-08-31

**Authors:** Yue-Xian Lin, Shu-Han Li, Wei-Chen Huang

**Affiliations:** 1 Department of Materials Science and Engineering, National Yang Ming Chiao Tung University, Hsinchu 30010, Taiwan; yuexlin118@gmail.com; 2 Undergraduate Honors Program of Nano Science and Engineering, National Yang Ming Chiao Tung University, Hsinchu 30010, Taiwan; u0711610.pn07@nctu.edu.tw; 3 Department of Electrical and Computer Engineering, National Yang Ming Chiao Tung University, Hsinchu 30010, Taiwan

**Keywords:** hydrogel neural interface, gelatin, microbial transglutaminase, transfer printing, adhesion

## Abstract

Hydrogels are the ideal materials in the development of implanted bioactive neural interfaces because of the nerve tissue-mimicked physical and biological properties that can enhance neural interfacing compatibility. However, the integration of hydrogels and rigid/dehydrated electronic microstructure is challenging due to the non-reliable interfacial bonding, whereas hydrogels are not compatible with most conditions required for the micromachined fabrication process. Herein, we propose a new enzyme-mediated transfer printing process to design an adhesive biological hydrogel neural interface. The donor substrate was fabricated via photo-crosslinking of gelatin methacryloyl (GelMA) containing various conductive nanoparticles (NPs), including Ag nanowires (NWs), Pt NWs, and PEDOT:PSS, to form a stretchable conductive bioelectrode, called NP-doped GelMA. On the other hand, a receiver substrate composed of microbial transglutaminase-incorporated gelatin (mTG-Gln) enabled simultaneous temporally controlled gelation and covalent bond-enhanced adhesion to achieve one-step transfer printing of the prefabricated NP-doped GelMA features. The integrated hydrogel microelectrode arrays (MEA) were adhesive, and mechanically/structurally bio-compliant with stable conductivity. The devices were structurally stable in moisture to support the growth of neuronal cells. Despite that the introduction of AgNW and PEDOT:PSS NPs in the hydrogels needed further study to avoid cell toxicity, the PtNW-doped GelMA exhibited a comparable live cell density. This Gln-based MEA is expected to be the next-generation bioactive neural interface.

## 1. Introduction

Implanted neural interfaces are the new-generation pharmaceutical implants that can permit ultrafast neuromodulation to improve the treatment of numerous neurological diseases [1,2]. Integrated with rigid and inorganic electrode materials, the implanted devices tend to induce inevitable foreign tissue responses, followed by chronic inflammation lasting for weeks to months, which finally disrupts the chronic signal transduction [3,4]. Flexible electronics have been developed using polymeric materials such as conductive polymers [5,6], parylene C [7,8], polyimide [9,10], or PDMS [11,12] to alleviate the mechanical damage induced by the rigid devices [13,14]. However, the sustained mechanical and biological mismatch between the device and nerve tissues still limits the long-term device function.

To suppress chronic inflammation and neuronal loss, much effort has been made on the design of “bioactive interfaces” with the construction of a device surface with native physical, chemical, and biological properties, mimicking that of biological tissues [15,16,17]. Recent studies even highlight a trend to develop a “living electrode” through embedding living cells on the electrode surface to offer both functions of neuromodulation and nerve tissue engineering [18]. Under these circumstances, hydrogels receive much attention because of the microstructural and mechanical properties mimicking both extracellular matrix and nerve tissues, whereas the 3D polymeric architecture with abundant ionized moieties can serve as a robust delivery vehicle for bioactive molecules [19,20]. The MEA covered with hydrogels made of bioactive macromolecules, such as collagen [21], chitosan [22,23], heparin, or silk [24], were found to enhance neuron attachment and neurite outgrowth, leading to an improvement in the chronic inflammation and device function [25,26]. However, the generally used coating techniques to integrate hydrogels and the device lead to non-reliable interfacial bonding between the hydrated polymer rigid/dehydrated electronic microstructure, that deteriorates easily over time [27,28]. In addition, because the swollen hygroscopic polymer networks are susceptible to degradations, hydrogels are fundamentally incompatible with almost all of the micromachining processing conditions.

Transfer printing allows for the assembly of components in a broad range of geometries or configurations [29,30]. We previously reported an aqueous phase-mediated transfer printing technique using swollen poly(ethylene glycol)(PEG)-based hydrogels with network precursors that contain catechol motifs to enhance interfacial adhesion in moisture and enable accelerated in-situ sol-gel phase transition [31]. This PEG-based MEA permitted reliable seamless adhesion on the peripheral nerve for neuronal signal recording, however, the inevitable oxidation of catechols leads to the gradual loss of ductility and adhesion of the hydrogels [32], which remains the challenge for maintaining the stability of long-term neural interfacing.

Herein, we propose a new enzyme-mediated transfer printing process that enabled the formation of an adhesive gelatin-based bioactive neural interface. The concept was inspired by gelatin (Gln) and a biological enzyme, microbial transglutaminase (mTG), that can catalyze the formation of the amide bond between the γ-carboxamide group of Gln [33] to permit both the gelation of Gln itself and the interfacial adhesion between the as-formed Gln-mTG hydrogels and the Gln-based electrode features. Photo-crosslinking of gelatin methacryloyl (GelMA) containing various conductive nanoparticles (NPs), including Ag nanowires (NWs), Pt NWs, and PEDOT:PSS, led to the formation of stretchable bioelectrodes as donor substrates, called NP-doped GelMA, with tissue-mimicked structural/mechanical properties and electrical conductivity. The receiver substrate-composed mTG-incorporated Gln exhibited temporally controlled gelatin and adhesion that permitted transfer printing of prefabricated NP-doped GelMA microelectrode arrays (MEA). The integrated hydrogel MEA were mechanically and structurally bio-compliant, while being stable in moisture to support the growth of neuronal cells, which is expected to be a next-generation bioactive neural interface.

## 2. Materials and Methods

### 2.1. Preparation and Characterization of Conductive NP-Doped GelMA

GelMA was synthesized by Bae Hoon Lee’s method [34]. Briefly, 100 mL of PBS was heated to 50 °C, followed by the addition of 10 g of Gln. After gln was dissolved in PBS, the solution pH was adjusted to 7.8, followed by the addition of 0.167 mL of methacrylic anhydride (MAA). The sequential loading of MAA (0.167 mL at each step) after pH adjustment every 30 min at 50 °C for 3 h was applied, and the solution pH of the PBS was monitored and readjusted to 7.8 every 30 min. Then, the homogeneous solution was shifted into a dialyzing membrane for dialysis. Deionized water was changed at intervals of 12 h for 7 days. The final product was obtained after freeze-drying in a lyophilizer for 4 days and kept at −20 °C.

On the other hand, three types of conductive particles were prepared as follows: Poly(ethylenedioxythiophene):poly(styrenesulfonate) (PEDOT:PSS) aqueous solution was purchased from Guang Sheng Material Co., Ltd., with the concentration adjusted to 10% *w*/*v* in water. Ag NWs were synthesized following Xia’s method [35], a glass vial containing 5 mL of ethylene glycol was heated at 150 °C for 1 h, followed by the addition of 40 μL of 4 mM CuCl_2_, then the mixture was heated for another 15 min. Then, 1.5 mL of 0.147M PVP solution in ethylene glycol was added into the heated mixture, followed by the dropwise injection of 1.5 mL of 0.094M AgNO_3_ solution in ethylene glycol under stirring. After AgNO_3_ was added, the stirrer was removed, and the glass vial was moved to a hydrothermal autoclave reactor. After 4 h of heating at 150 °C, the product was washed three times with acetone and finally resuspended in water with 10% *w*/*v*. For the synthesis of Pt NWs [36], a solution containing 4 mL of ethylene glycol (EG) and 6 mL of N,N-dimethylmethanamide (DMF) was added into 500 mg of KOH mixed with 300 mg of H_2_PtCl_6_. After stirring for 12 h, the solution was transferred into a 25 mL Teflon autoclave and maintained at 170 °C for 8 h. After cooling, the precipitated black products were formed and washed with ethanol three times.

NP-doped GelMA hydrogels were prepared by adding GelMA powders into 10% *w*/*v* NPs (PEDOT:PSS, AgNWs, PtNWs) water solution to obtain the final concentration of GelMA as 10% *w*/*v*. The homogenous mixture was added with 1% *w*/*v* photo-initiator (Irgacure 2959, Sigma-Aldrich). The final solution was casted and cross-linked by applying UV light (OmniCure S2000 UV lamp, 20 W/cm^2^ 30 s) for gelation. The morphology of hydrogels was observed using scanning electron microscopy (SEM, JEOL-JSM6700) with an accelerating voltage of 5 kV. Stress–strain measurements were performed by uniaxial testing on the as-prepared hydrogels by MTS Tytron 250. Each dry sample was cut into a dog-bone shape of 3 cm in length and 1 cm in width. The applied load was set in a window range from 0 to 50 N with a working rate of 0.1 mm/s. The electrochemical property of the NP-doped GelMA hydrogels was determined by electrochemical impedance spectroscopy (EIS) using the electrochemical instrument 600E Potentiostat/Galvanostat (CH Instruments, Inc., Austin, TX, USA), with a standard three-electrode system (an indium tin oxide (ITO) glass substrate coated with hydrogel samples in an area of 1 × 1 cm as a working electrode, a platinum wire as a counter electrode, and an Ag/AgCl working electrode) in 0.1M PBS. Impedance response was assessed by the application of amplitude sinusoids of 100 mV across the frequency range of 1 Hz to 1 MHz. The mean impedance magnitude was presented on a Bode plot and a Nyquist plot obtained by the average of three scans for each sample. For cyclic voltammetric tests, the scan rate was 50 mV/s and the applied potential window was set as −0.8 to 0.8 V with 50 cycles in scan number.

### 2.2. Preparation and Characterization of Gln-mTG

Gln-mTG hydrogels were prepared based on the weight ratios of [mTG]:[gelatin] of 0:75, 2:75, and 8:75 in 75 mg/mL of gelatin water solution. The gelation dynamics were measured indirectly using rheometry in a parallel plate geometry. Freshly prepared precursor solutions were applied to the center of the plate with a nominal gap distance of 800 µm. The storage (G′) and loss (G″) moduli were measured by a linear time sweep (strain amplitude of γ = 1%; angular frequency of ω = 1 Hz for 30 min). The mechanical properties of Gln-mTG hydrogels were measured by uniaxial stress–strain measurements. Rectangular samples (1.5 × 10 mm^2^) were strained uniaxially at 2 mm min^−1^ to obtain stress–strain curves. To measure the degradation ratios and swelling ratios changing over time, Gln-mTG hydrogels were pre-weighed (W_0_) after gelation, then the samples were immersed in PBS at 37 °C. The samples were taken out at intervals of 15 min for 1 h to weigh again (W_1_). The swelling ratios of the hydrogels were calculated gravimetrically using Equation (1):

(1) $$SR=\frac{W_{1}-W_{0}}{W_{0}}\times 100\%$$

where *W*_1_ and *W*_0_ correspond to the mass of swollen and original hydrogels, respectively (n = 5). Interfacial adhesion was measured between freshly prepared Gln-mTG hydrogels and the indenter. Hydrogels with cylindrical form factors (D × h = 20 × 2 mm) were mounted on the platform. Indenters with flat cylindrical glass windows (D = 5 mm; Edmund Optics, Barrington, NJ, USA) were attached to a vertical motorized stage for indentation, with the measured loads at a 1 kHz sampling rate, the indenter with a constant preload force of 50 ± 5 mN for the contact with the hydrogels for 5 min, followed by retracted at a constant speed of 1 mm s^−1^, and then the force–distance curves were recorded.

### 2.3. Fabrication of Hydrogel Microelectrode Arrays (MEA)

**Figure 1:**
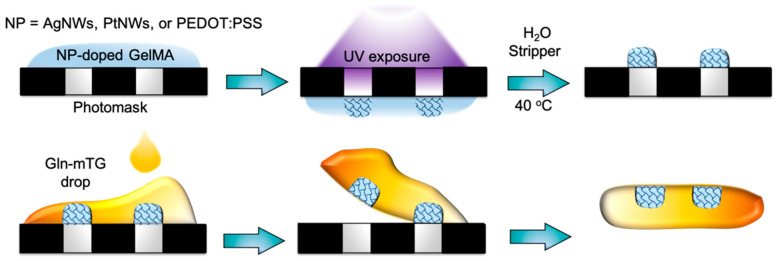
The fabrication process flow of hydrogel-based MEA.

The schematic drawing of the fabrication process flow is shown in Figure 1, where the homogeneously distributed NP-doped GelMA solution prepared by ultrasound treatment was spin-coated on the photomask to obtain the thickness of 10–20 µm, followed by treatment with UV for crosslinking. After immersing in water at 40 °C to remove the un-crosslinked residues, the final patterns were obtained. To transfer print NP-doped GelMA hydrogel MEA from the photomask (donor substrate) to a Gln-mTG substrate (target substrate), the freshly prepared Gel-mTG precursor solution was dropped on the as-formed patterns. After 1 h of gelation, the hydrogel was retrieved, and the microelectrode arrays were transfer printed onto Gln-mTG.

### 2.4. Characteristics of the Fabricated Hydrogel MEA

The topography and morphology of the as-fabricated hydrogel devices were observed by optical microscope and SEM. The stretchability of the hydrogel devices was investigated by uniaxial testing using MTS Tytron 250 to show the stress–strain profiles. The conductivity of NP-doped GelMA circuits was expressed by resistance obtained from the cyclic I-V curves constructed using the CHI 614C electrochemical workstation (CHI Instruments, Austin, TX, USA). For the three-electrode system cell configuration, the working voltage window ranged from −0.8 to 0.8 V, with two-point contact by the working electrode and counter electrode at a distance of 5 mm, and the signal controlled by the Ag/AgCl reference electrode was returned via a platinum (Pt) counter electrode.

### 2.5. In Vitro Cell Responses to Hydrogel Devices

PC12, a cell line derived from a pheochromocytoma of the rat adrenal medulla, was used for the in vitro study. The cells were seeded on samples with DMEM/F12 medium (Gibco Dulbecco’s Modified Eagle Medium: Nutrient Mixture F-12, Thermo Fisher Scientific, USA), supplemented with 10% fetal bovine serum and 1% antibiotic-antimycotic (Gibco anti-anti, Thermo Fisher Scientific, Waltham, MA, USA), and incubated in a humidified chamber set at 37 °C in 5% CO_2_. Cytotoxicity was explored using CytoSelectTM Cell viability and the cytotoxicity assay (Cell Biolabs, Inc., San Diego, CA, USA). The Live–Dead reagents include calcein-AM for staining the living cells (green) and ethidium homodimer-1 (EthD-1) to stain the dead cells (red). The cells were seeded on the substrates, including GelMA, AgNW-doped GelMA, PtNW-doped GelMA, and PEDOT:PSS-doped GelMA, with 1 × 1 cm in area at 1 × 10^5^ cells/well. After 24 h cell culture, the diluted Live–Dead reagents in PBS were directly added to the cell culture media at the ratio of reagent to culture media of 1:1, followed by a gentle mixing. After incubation in the dark for 30 min, five representative fields of view were selected per well under fluorescent microscope. Numbers of live (green) and dead (red) cells were counted using the multi-wavelength cell scoring module of MetaMorph software. All data are reported as mean ± standard deviation (SD) of the tests performed in triplicate. Statistical analysis was carried out by one-way ANOVA followed by Student’s *t* test to determine statistical significance (*p* < 0.05).

## 3. Results and Discussion

### 3.1. Characteristics of Conductive Biological Tissue-Mimicked NP-Doped GelMA Composites

**Figure 2:**
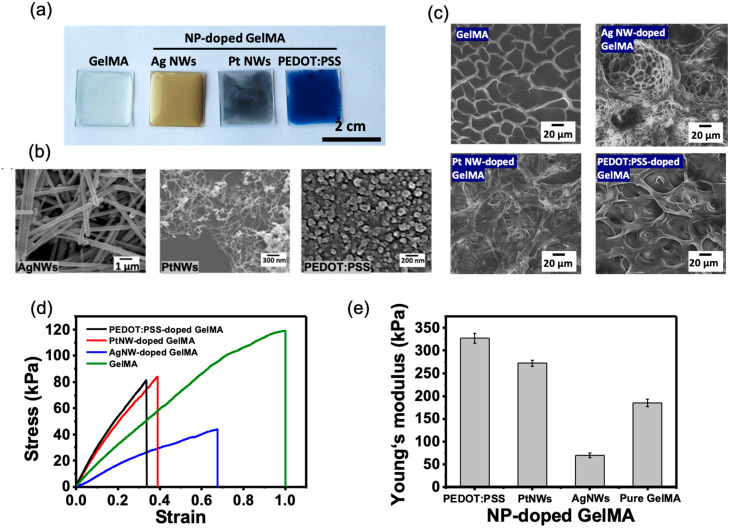
The structural and mechanical properties of NP-doped GelMA. (**a**) The appearance and color of NP-doped GelMA thin films. (**b**) SEM images of the morphology of each NP. (**c**) SEM images showing the porous structure of NP-doped GelMA hydrogels. (**d**) Stress–strain curves of GelMA and NP-doped GelMA. (**e**) The calculated Young’s modulus of each sample.

Light-induced polymerization of GelMA permits the immobilization and the connection of the incorporated conductive NPs to form three-dimensional conductive hybrid hydrogels. Figure 2a–c show the structural properties of the NP-doped GelMA. The color of the GelMA, AgNW-doped GelMA, PtNW-doped GelMA, and PEDOT:PSS-doped GelMA hybrid films was observed to be transparent, yellow, dark, and blue, respectively. The SEM image in Figure 2b shows the morphology of AgNWs, PtNWs, and PEDOT:PSS NPs, where AgNWs are ~500 nm in width and 25–50 µm in length, PtNWs are ~10 nm in width and 5–15 µm in length, and PEDOT:PSS are ~50–100 nm in diameter. All NP-doped hydrogels show different porous microstructures in the SEM images (Figure 2c). Particularly, the pristine GelMA and PEDOT:PSS-doped GelMA demonstrated a smooth pore wall with a pore size distribution ranging from 10 to 50 µm, while the AgNW- and PtNW-doped GelMA exhibited a filamentary microstructure with a pore size as small as 1 µm. The mechanical properties of hydrogels were measured by uniaxial testing. As shown in the obtained stress–strain profiles in Figure 2d, with the addition of NPs, the tensile strain was reduced because of the incorporated metallic or rigid NPs that limited the ductility of GelMA. However, the incorporation of NPs is able to increase surface energy that can improve the elasticity, and it can be seen in Figure 2e that PEDOT:PSS-doped and PtNW-doped GelMA demonstrated higher young’s modulus than that of the pristine GelMA. The lower Young’s modulus found from AgNW-doped GelMA may be due to the relatively larger size of AgNWs than those of the other NPs which hinder UV absorption during hydrogel fabrication, thereby resulting in the reduced crosslinking degree of GelMA [37].

**Figure 3:**
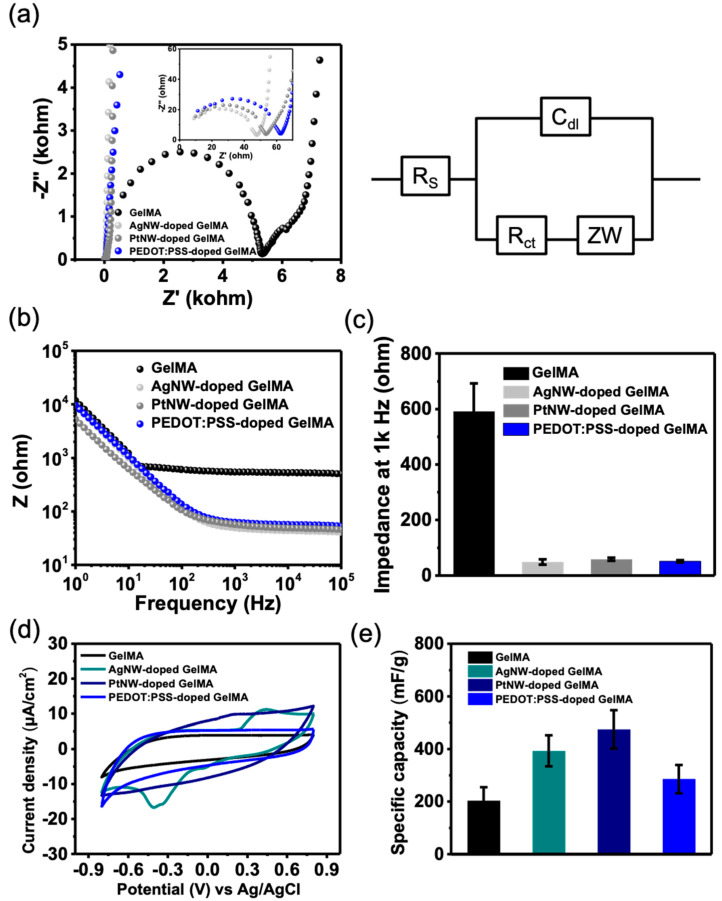
Electrochemical properties of NP-doped GelMA. (**a**) Nyquist diagram and the corresponding equilibrium circuit model. (**b**) Bode plot showing the impedance modulus over frequencies. (**c**) The impedance values at 1 kHz. (**d**,**e**) The CV curves and the calculated specific capacity of the hydrogels.

The electrochemical impedance measurement of all hydrogels is demonstrated in Figure 3. In the Nyquist diagram in Figure 3a, all the profiles can be explained by a proposed equivalent circuit model of constant phase element, i.e., Randles circuit, that represents the double-layer capacitor behavior of the rough electrodes [38]. The NP-doped GelMA showed the significantly smaller semicircles as compared with pure GelMA, indicating the significant reduction of ionic and electrical resistance. The corresponding Bode plot in Figure 3b shows that the NP-doped GelMA demonstrates a lower impedance value than that of GelMA over all the frequency values. The performance of neural recording electrodes is typically evaluated by their impedance at 1 kHz [39,40]. At 1 kHz, the measured impedance value for GelMA was 592 ± 22.7 Ω, while the NP-doped GelMA showed a significantly lower impedance, ranging from 38.3 to 52.4 Ω (Figure 3c). The cyclic voltammogram (CV) in Figure 3d demonstrates that the incorporation of NPs contributed to the increase of the enclosed area of CV curves, indicative of the electroactive areas provided by NPs increasing the charge storage capacity (Figure 3e).

### 3.2. Characteristics of Gel-mTG Hydrogels

**Figure 4:**
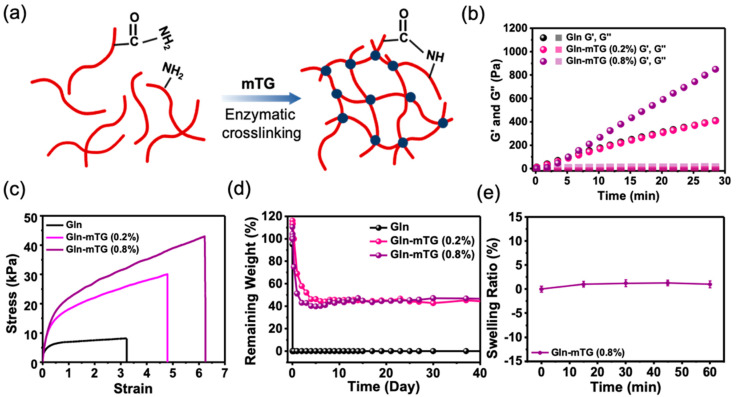
(**a**) A schematic drawing demonstrating that the mTG catalyzes the chemical crosslinking that leads to the formation of mechanically robust Gln hydrogels. (**b**) The storage and loss modulus, G′ and G″ respectively, versus time of Gln with different concentration of mTG. (**c**) The stress–strain curves of Gln and Gln-mTG. (**d**) The degradation profiles showing the remaining weight over time. (**e**) The swelling ratio of Gln-mTG (0.8%) over time.

When the glutamine and lysine residues of Gln are exposed to microbial transglutaminase (mTG), the enzyme catalyzes the formation of amide bonds, giving rise to the chemical crosslinking that leads to the formation of a mechanically robust hydrogel (Figure 4a). The gelation kinetics and the mechanical properties of the resultant hydrogels are controllable by the mTG concentration. The storage modulus−time behavior is shown in Figure 4b, where the G′ of the hydrogels is increased at a constant speed after the addition of mTG. The hydrogel prepared with 0.8% mTG produced elastic networks with a G′ of 847 ± 21 Pa within 30 min. The bulk mechanical properties of the pristine Gln and Gln-mTG hydrogels were measured by uniaxial tensile testing (Figure 4c). Table 1 shows the measured and calculated mechanical properties of all hydrogels, where all the strain, Young’s modulus, tensile strength, and toughness values improved as the mTG concentration increased. Compared with pristine Gln, the Gln-mTG containing 0.8% mTG exhibited a ~2-fold, 3-fold, 5-fold, and 9-fold increase in strain, Young’s modulus, tensile strength, and toughness, respectively. When immersed in water, the pure Gln hydrogel showed rapid degradation in water within 30 min, while the Gln-mTG hydrogels enabled a sustained structure integrity until three months (Figure 4d). In addition, the Gln-mTG containing 0.8% mTG showed almost no swelling in water, indicating that the hydrogel is not easily deformed when being hydrated (Figure 4e).

### 3.3. Transfer Printing NP-Doped Gln Microelectrode Arrays onto Gln-mTG Hydrogels

**Figure 5:**
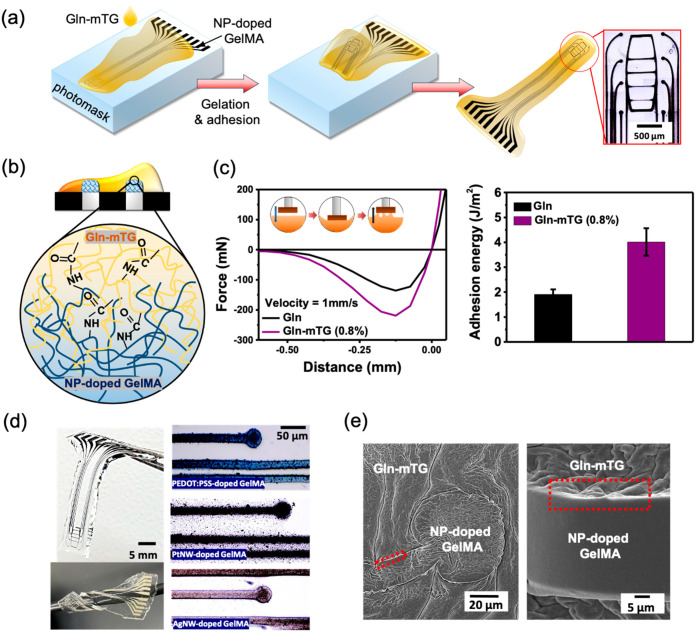
Transfer printing fabrication of Gln hydrogel MEA. (**a**,**b**) Schematic representation illustrating the mechanisms that enable one-step transfer printing microelectrode patterns of NP-doped GelMA to Gln-mTG hydrogels. (**c**) Representative force–distance curves obtained by retracting the indenter from the surface of Gln and Gln-mTG. (**d**) The naked views of the as-fabricated adhesive hydrogel MEA, which entangles around a wire with 3 mm in diameter. Optical images showing the enlarged views of NP-doped GelMA microelectrodes patterning on the Gln-mTG. (**e**) Scanning electron microscopic images of the hydrogel MEA showing continuous interfaces in the bonding area.

The mTG enzyme in gelatin exhibits covalent bond-enhanced adhesion to NP-doped GelMA, permitting the encapsulation of NP-doped GelMA microelectronic structural features on the Gln-mTG hydrogels. As shown in Figure 5a, the NP-doped GelMA 8-channel electrodes and the traces were directly formed on the photomask through photolithography. Without the application of a sacrificial layer, the in situ gelation Gln-mTG hydrogels can produce conformal contact and robust adhesion with the underlying NP-doped GelMA features, in turn forming a fully hydrogel-comprised microelectronic device. The key factor to the success of this simple transfer printing relies on the formation of amide bonds that result in the simultaneous mTG-induced gelation and mTG-induced interfacial adhesion (Figure 5b). The adhesion of Gln and Gln-mTG hydrogels was measured via recording force–distance curves. The representative force–distance curves in Figure 5c indicated that a larger force was required to detach the indenter from the Gln-mTG hydrogel, as compared with the pure gelatin. The adhesive energy is expressed by the effective tensile work, W_ten,eff_, in Figure 5d, showing that the W_ten,eff_ of Gln-mTG hydrogels was two times higher than that of gelatin. Figure 5d shows the appearance of the as-fabricated device. With the mTG-induced adhesion, the device is able to seamlessly entangle around a wire with 3 mm in diameter. The optical image shows the as-transferred NP-doped GelMA micropatterns with a resolution down to 30 µm. The SEM images in Figure 5e show the morphology of the device microstructure, where no clear interface was visible in the bonding area, revealing a robust adhesion between the layered interface.

### 3.4. Conductivity of Stretchable Gln-Based Hydrogel Devices

**Figure 6:**
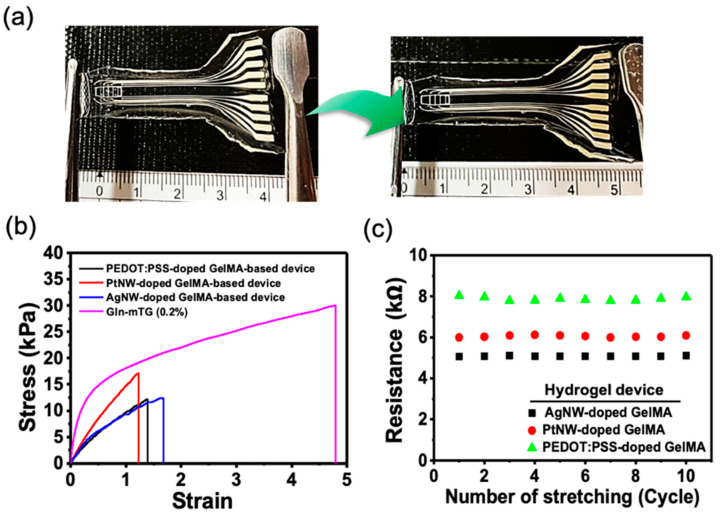
Conductivity of stretchability of Gln hydrogel MEA. (**a**) Naked views showing the stretch property of the device. (**b**) The stress–strain curves of Gln and the fabricated device. (**c**) Cyclic resistance measurement of the NP-doped GelMA circuits at stretched and relaxed states.

Gln-based hydrogel MEA are deformable and stretchable, with stable conductivity. Uniaxial tensile tests were performed on the Gln-mTG and the fabricated Gln-based MEA devices. As demonstrated in the image in Figure 6a, with the uniaxial stretch of the whole device, there is no delamination of MEA micropatterns from the underlying Gln-mTG, indicating a stable adhesion of the layered structure. The stress–strain curves are shown in Figure 6b. The Gln-mTG hydrogels achieved maximum tensile strain of 4.88 ± 1.21. In contrast, the integrated MEA showed a smaller strain, with values ranging from 1.2 to 1.8. The resistance of the as-transferred MEA was measured by two-probe I-V measurements. As shown in Figure 3c, the calculated end-to-end resistance remained constant for 10 stretching cycles of ~20% elongation. In the meantime, the AgNW-doped GelMA electrodes showed the lowest resistance, which showed the correspondence to the measured impedance shown in Figure 3b.

### 3.5. Cell-Laden Capacity and Cytotoxicity of Gelatin-Based Hydrogel Devices

**Figure 7:**
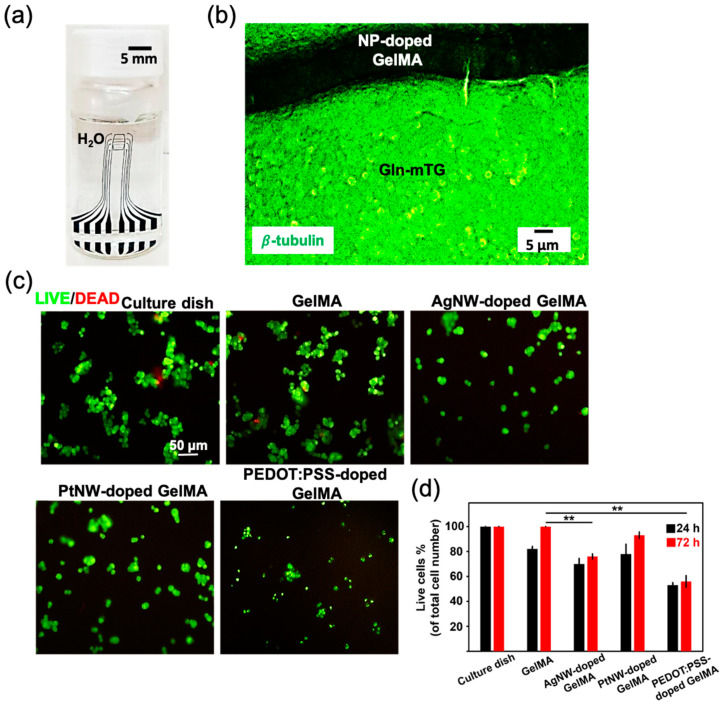
(**a**) An optical image demonstrating no morphology change of the hydrogel device. (**b**) A fluorescence image of PC12 cell differentiation on the hydrogel device after 7 days. (**c**) Live–Dead assay of the cells was performed after 72 h. (**d**) Percentage of survived cells as calculated under a fluorescence microscope (* indicates significant difference in different groups for comparison, ** *p* < 0.01 as compared with GelMA group).

Figure 7a shows the appearance of the hydrogel device immersed in water for 1 month, confirming that the device preserved structural stability after hydration. Accordingly, the neural interface compatibility of the hydrogel devices was explored via investigating cell adhesion and cytotoxicity. After 7 days of cell differentiation, it was found on the fluorescent image in Figure 7b that PC12 cells were able to show adhesion and differentiation on the whole device. Concerned about the effect of the incorporated NP on the cell activity, the Live–Dead assay was performed for the cells on the GelMA and NP-doped GelMA. After 24 h of cell culture, the survived cells stained in green were clearly observed on the fluorescent images in Figure 7c. There were few cells in red to be observed due to the detachment after cell death. The statistic percentage of survived cells is demonstrated in Figure 7d, where GelMA showed a similar live cell density to that of the culture dish. As compared with GelMA, PtNW-doped GelMA exhibited a comparable live cell density, while a significant decrease in live cell number was observed for the groups of AgNW-doped GelMA and PEDOT:PSS-doped GelMA. Such result fits well with the commonly recognized concept that Pt exhibits robust biocompatibility for implantation [41], while Ag is biologically toxic [41]. The decrease of living cells on PEDOT:PSS-doped GelMA may be attributed to the toxicity of the surfactant PVP in the commercialized PEDOT:PSS solution. Overall, the results indicate that the gelatin-based hydrogel device enables cell growth to be a potential bioactive neural interface.

## 4. Conclusions

A biological hydrogel bioelectronic device was fabricated by enzyme-mediated transfer printing of NP-doped GelMA bioelectrodes to Gln-mTG hydrogels. Light-induced polymerization of GelMA is compatible for various incorporated conductive NPs, forming three-dimensional micropatterned bioelectrodes with both biological compliance and electrical conductivity. The use of mTG provided a covalently bonding interaction between the NP-doped GelMA electrode features and Gln hydrogels, leading to the formation of Gln-based hydrogel MEA with layered stability in moisture for cell adhesion and growth. Much effort is needed to develop an ideal neural implant with complex multilayered stacks. In addition, the introduction of conductive NPs needs further study, and the NPs should be prepared carefully to avoid cell toxicity. Nevertheless, the enzyme-mediated transfer printing requires no sacrificial layer, toxic agents, or complicated serial micromachine process, which provides an opportunity for the rapid fabrication of hydrogel bioelectronics.

## Figures and Tables

**Table 1:** Mechanicial Properties obtained from stress-strain curves of Gln and Gln-mTG hydrogels.

	Strain	Young’s Modulus (kPa)	Tensile Strength (kPa)	Toughness (kJ/m^3^)
**Gln**	3.4 ± 0.8	58.30 ± 3.21	8.13	22.79
**Gln-mTG (0.4%)**	4.7 ± 0.2	173.12 ± 10.61	30.06	107.05
**Gln-mTG (0.8%)**	6.2 ± 0.7	180.95 ± 9.22	43.02	191.21
